# Synergistic multi-joint kinematic strategies to reduce tripping risks during obstacle-crossing in older long-term Tai-Chi Chuan practitioners

**DOI:** 10.3389/fnagi.2022.961515

**Published:** 2022-09-29

**Authors:** Hsing-Po Huang, Chien-Chung Kuo, Shiuan-Huei Lu, Sheng-Chang Chen, Tsung-Jung Ho, Tung-Wu Lu

**Affiliations:** ^1^Department of Biomedical Engineering, National Taiwan University, Taipei, Taiwan; ^2^Department of Orthopedics, China Medical University Hospital, Taichung, Taiwan; ^3^Department of Orthopedics, School of Medicine, China Medical University, Taichung, Taiwan; ^4^Integration Center of Traditional Chinese and Modern Medicine, Buddhist Tzu Chi General Hospital, Hualien, Taiwan; ^5^Department of Chinese Medicine, Buddhist Tzu Chi General Hospital, Hualien, Taiwan; ^6^School of Post-baccalaureate Chinese Medicine, Tzu Chi University, Hualien, Taiwan; ^7^Department of Orthopaedic Surgery, School of Medicine, National Taiwan University, Taipei, Taiwan

**Keywords:** Tai-Chi Chuan, kinematics strategies, balance control strategies, obstacle-crossing, fall risk

## Abstract

**Introduction:**

Losing balance or tripping over obstacles is considered one of the most common causes of falls in the elderly. Tai-Chi Chuan (TCC) has been shown to improve muscle strength, inter-joint coordination and balance control in the elderly. This study aimed to determine whether older long-term TCC practitioners would show multi-joint kinematic strategies that would reduce the risk of tripping during obstacle-crossing compared to peers without TCC experience.

**Methods:**

Three-dimensional motions of the pelvis and lower extremities were measured using a motion capture system in fifteen older long-term TCC practitioners (TCC group) and 15 healthy controls without TCC experience during walking and crossing obstacles of three different heights. Crossing angles of the pelvis and lower limbs and toe-obstacle clearances were obtained and analyzed using two-way analyses of variance to study the between-subject (group) and within-subject (height) effects. A multi-link system approach was used to reveal the relationship between joint angular changes and toe-obstacle clearances.

**Results:**

Compared to the controls, the TCC group showed increased leading and trailing toe-obstacle clearances (*p* < 0.05) with increased pelvic hiking and hip flexion but decreased hip adduction on the swing side and decreased knee flexion on the stance side during leading-limb crossing (*p* < 0.05), and increased pelvic hiking and anterior tilt but decreased hip adduction on the swing side, and decreased knee flexion on the stance side during trailing limb crossing (*p* < 0.05). All significant joint angular changes contributed to the increases in the toe-obstacle clearances.

**Conclusion:**

The current study identified the kinematic changes of the pelvis and the lower limb joints and revealed a specific synergistic multi-joint kinematic strategy to reduce tripping risks during obstacle-crossing in older long-term TCC practitioners as compared to non-TCC controls. The observed multi-joint kinematic strategies and the associated increases in toe-obstacle clearances appeared to be related to the training characteristics of TCC movements. Long-term TCC practice may be helpful for older people in reducing the risk of tripping and the subsequent loss of balance.

## Introduction

More than one-third of people aged 65 and over fall at least once during a year (Tinetti and Kumar, 2010; Verma et al., 2016) and fall death rates are the highest among adults over the age of 60, according to the World Health Organization (World Health Organization [WHO], 2021). Factors affecting fall risks include intrinsic factors related to the ability of balance, such as vision, proprioception, vestibular system, musculoskeletal system, and sensorimotor functions (Sturnieks et al., 2008) and extrinsic factors such as environmental hazards (Van Dieen et al., 2005), including the presence of obstacles, slippery surfaces, curbs, and stairs (Tinetti et al., 1988; Tinetti and Kumar, 2010). An estimated 60–67% of falls in the elderly occur inside the home or in immediate surroundings (Stevens et al., 2014; Crenshaw et al., 2017) and often involve environmental hazards (Letts et al., 2010). Losing balance or tripping during obstacle-crossing is one of the most common causes of falls in the elderly (Chou et al., 2001; Menant et al., 2010). For successful obstacle-crossing, it is essential to maintain the body’s stability while lifting the swing foot to clear the obstacle (Chen et al., 2008; Huang et al., 2008). Obstacle-crossing thus requires lower-limb strength (stance foot), precise end-point (swing foot) control and highly coordinated joint movements while maintaining body balance (Chen et al., 2004; Chen and Lu, 2006; Lu et al., 2006). Any inappropriate control of the locomotor system may affect the inter-joint and joint-to-endpoint kinematic coordination, leading to tripping over obstacles or body imbalance (Draganich and Kuo, 2004; McKenzie and Brown, 2004; Lu et al., 2006; Liu et al., 2010; Hsu et al., 2016). Since obstacles are an unavoidable part of our daily living, it is essential to improve the intrinsic fitness of older people to reduce fall risks while negotiating obstacles during daily activities (Bueno-Cavanillas et al., 2000; Prevention and Panel, 2001; Ikezoe et al., 2003).

Exercises such as walking, jogging, cycling, table tennis, resistance training, weight-bearing exercise, and Tai-Chi Chuan (TCC) are beneficial for older people to improve their general fitness and health (Martyn-St James and Carroll, 2008; Peterson et al., 2010; Oja et al., 2011; Kim et al., 2014; Ikenaga et al., 2017; Naderi et al., 2018; Pasqualini et al., 2019; Izquierdo et al., 2021). Among these exercises, TCC, a low-speed and low-impact ancient Chinese martial art, is becoming popular among the elderly for improving their general physical condition. TCC is an effective multi-factor exercise (Shubert et al., 2010; Bird et al., 2011), which has been shown to improve multiple intrinsic factors and the general mental and physical function in the elderly (Wu, 2002; Tsang et al., 2004; Mao et al., 2006; Ho et al., 2012; Okuyan Birimoğlu and Bilgili, 2017; Liu et al., 2019; Birimoglu Okuyan and Deveci, 2021). TCC consists of a series of slow, continuous, and gentle motions transitioning between single-limb support and double-limb, focusing on the dynamic stability of the stance limb and precise control of the swing limb and the weight shift between limbs. Through regular and prolonged TCC practice, one could increase muscle strength (Wu et al., 2002; Mao et al., 2006), flexibility (Lan et al., 2008; Huang and Liu, 2015), balance (Wu, 2002; Mak and Ng, 2003; Huang and Liu, 2015), inter-joint coordination (Wang et al., 2010a; Kuo et al., 2021b), and sensory organization in postural control (Tsang et al., 2004). Long-term TCC practice could also attenuate the age-related decline in general physical function and lead the practitioners to modify their gaits and movement patterns (Wolf et al., 1997; Lin et al., 2006; Mak et al., 2017). Furthermore, previous studies have shown that TCC training could have positive effects on posture, gait, and movement performance, such as improvement of standing balance (Ho et al., 2012) and walking performance (Hackney and Earhart, 2008; Li et al., 2012, 2014). Such benefits in TCC practice might reduce the risk in the elderly, which could happen during daily locomotion in diversified environments, e.g., obstacle negotiation.

During level walking, the locomotor system (i.e., the pelvis-leg apparatus) acts as a multi-link system with complex yet coordinated movements (Saunders et al., 2005; Franz et al., 2009). These movements become more complex when individuals encounter obstacles or negotiate uneven terrains (Wang, 2003). Stepping over an obstacle changes the repetitive inter-joint movement patterns during level walking, placing greater challenges to the whole-body balance and end-point control with an increased risk of falling (Wang, 2003). With the pelvis-leg apparatus as a multi-link system, a change in the angle of a joint leads to angular changes at other joints, which together determine the end-point position of the swing limb. Such inter-joint and joint-to-end-point kinematic relationships can be synthesized from the kinematic changes at individual joints and the end-points to identify the kinematic strategies of obstacle-crossing in various populations (Hsu et al., 2016; Chien and Lu, 2017; Wu et al., 2019a). Recently, the whole-body balance control, lower-limb inter-joint coordination and the multi-objective optimal control of obstacle-crossing have been studied in older people with long-term TCC experience, showing better performance as compared to non-TCC peers (Kuo et al., 2021a,b, 2022). However, no study has reported the kinematic strategy of the pelvis-leg apparatus and its effects on the end-point position control in relation to the risk of tripping.

This study aimed to determine the kinematic strategies of the pelvis-leg apparatus during obstacle-crossing in older healthy people with long-term TCC experience as compared to the non-TCC healthy controls. It was hypothesized that older people who had practised TCC over a long time would have higher toe-obstacle clearance with a reduced risk of tripping *via* a specific kinematic strategy of the pelvis-leg apparatus compared to non-TCC peers.

## Materials and methods

### Study design

**FIGURE 1:**
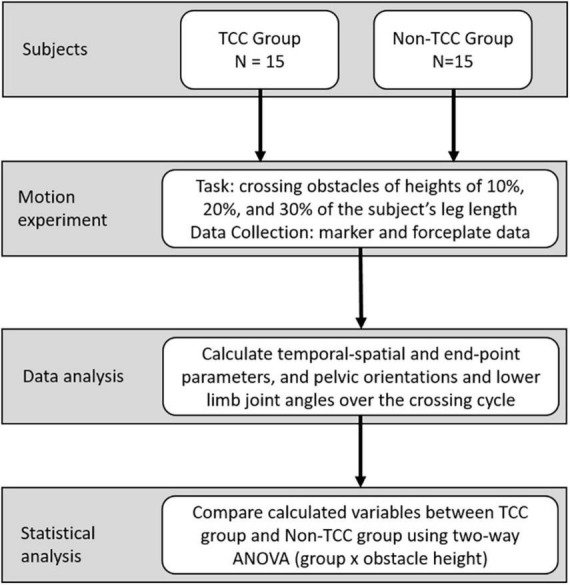
Fifteen long-term TCC practitioners and fifteen non-TCC practitioners were recruited for this study. All the practitioners were requested to cross the obstacles at three different heights during walking. At the same time, the three-dimensional motions of the pelvis and lower extremities were measured using a motion capture system. After data collection, joint angles of lower extremities and pelvic kinematics were calculated, and the data from the TCC group and Control group were compared.

The current observational cross-sectional study, which included participants aged over 65 years, was conducted by trained therapists in a university hospital gait laboratory from June 2009 to June 2010. The research methods consisted of the application of a validated skin marker-based motion capture system and forceplates to appraise temporal-spatial and end-point parameters and pelvic orientations and lower limb joint angles when crossing obstacles of three different heights (Figure 1). All the experiments and procedures conformed to the Ethical Principles for Medical Research Involving Human Subjects (World Medical Association Declaration of Helsinki). All subjects gave informed written consent as approved by the Institutional Review Board (IRB No. DMR98-IRB-072).

### Subjects

iFfteen healthy older adults with long-term TCC experience (TCC group) were recruited from a local TCC club to participate in the current study. Fifteen healthy controls without TCC experience but doing daily walking or jogging (Control group) were also recruited to match the TCC group for gender, age and BMI. The inclusion criteria for both groups: (1) aged 65 years and above, (2) active community-dwelling individuals, (3) physical independence in daily activities, (4) with normal or corrected vision, and (5) free from any neuromusculoskeletal injuries, disease or dysfunction that might affect gait and obstacle-crossing, such as ligament deficiency, joint degeneration, muscle weakness, vestibular deficiency, and stroke. Additionally, the TCC group should have practised TCC at least 60 min a day and 5 days a week for ten or more years, and the Control group should do walking or jogging daily. The exclusion criteria for both groups were: (1) with any cognitive impairments, (2) prior surgeries on the neuromusculoskeletal system, such as total joint replacements, (3) with cardiovascular or pulmonary diseases, and (4) fall experiences within a year at the time of the experiment.

### Experimental protocol

In a gait laboratory, each of the subjects walked at their preferred walking speed along a 10-meter walkway and crossed a tube-like obstacle placed horizontally across a height-adjustable frame located in the middle of the walkway (Chen and Lu, 2006). Two infrared-retroreflective markers placed on each end of the tube were also used to define the position and height of the obstacle. Thirty-nine infrared-retroreflective markers placed on specific anatomical landmarks were used to track the motions of the body segments, namely ASISs, PSISs, greater trochanters, mid-thighs, medial and lateral epicondyles, heads of fibulae, tibial tuberosities, medial and lateral malleoli, navicular tuberosities, fifth metatarsal bases, big toes and heels, and mandibular condylar processes, acromion processes, C7, medial and lateral humeral epicondyles, and ulnar styloid (Hong et al., 2015). Three-dimensional trajectories of the markers were measured using a seven-camera motion capture system (Vicon 512, Oxford Metrics Group, UK) at 120 Hz, while two regularly calibrated forceplates (AMTI, USA) (Hsieh et al., 2011) placed on either side of the obstacle were used to measure the ground reaction forces at 1080 Hz. The forceplate data were used to determine the gait events, including bilateral heel-strikes and toe-offs. The test conditions were crossing obstacles of 10, 20, and 30% of the subject’s leg length (i.e., the distance between the ASIS and medial malleolus) in a random order, with each lower limb leading (Chen and Lu, 2006). A 5-min break was allowed when changing obstacle-height conditions. A trial was defined as unsuccessful if the subject hit the obstacle during the crossing. In the current study, all the subjects could cross the obstacles successfully without hitting the obstacle. Data for three successful crossing trials for each lower limb leading were obtained for each obstacle height for each subject.

### Kinematic data analysis

With the measured marker data, temporal-spatial and end-point parameters were calculated. The crossing speed was calculated as the distance traveled by the mid-point of the ASIS markers in the walking direction divided by the time spent from the leading toe-off immediately before crossing to the trailing heel-strike immediately after crossing. The crossing stride of the leading limb began at toe-off immediately before the obstacle and ended at the subsequent toe-off, while that for the trailing limb was defined as heel-strike immediately before the obstacle to the subsequent heel-strike (Liu et al., 2018). Toe-obstacle clearances for both the leading and trailing limbs were calculated as the vertical distance between the toe marker of the swing limb and the obstacle when the toe marker was directly above the obstacle. The toe-obstacle distances were defined as the horizontal distance between the obstacle and the toe marker during stance immediately before stepping over the obstacle, while the heel-obstacle distances were defined as the horizontal distance between the obstacle and the heel marker during stance immediately after stepping over the obstacle (Wu et al., 2019a).

For the calculation of the orientations of the pelvis and the angles of the lower limb joints, each body segment was embedded with an orthogonal coordinate system with the positive *x*-axis directed anteriorly, the positive *y*-axis superiorly and the positive *z*-axis to the right following ISB recommendations (Wu and Cavanagh, 1995). The orientations of the pelvis were described relative to the laboratory coordinate system, with the leading limb as the reference limb. Pelvic hiking (drop) indicated that the hip was higher (lower) than the contralateral hip, while ipsilateral rotation indicated that the ipsilateral hip was anterior to the contralateral hip (Wu et al., 2019b). A Cardanic rotation sequence of z-x-y was used to calculate the angles of each lower limb joint (Grood and Suntay, 1983). The calculated angles when the leading and trailing toes were above the obstacle, called crossing angles (Lu et al., 2006), were extracted for subsequent statistical analysis. Effects of soft tissue artifacts of the pelvis-leg apparatus were reduced using a global optimization method (Lu and O’connor, 1999).

### Statistical analysis

For statistical comparisons between TCC and Control, independent *t*-tests were used for the demographic data, while a two-way mixed-design analysis of variance (ANOVA) was used for the temporal-spatial and end-point parameters, and all the calculated kinematic variables with one between-subject factor (group) and one within-subject factor (obstacle height). For all the statistical analyses, data of each calculated variable from both sides were averaged before further averaged across trials for each subject. All the calculated variables were of normal distribution determined by a Shapiro–Wilk test, and Levene’s test confirmed the homogeneity of variance across groups. In the absence of significant interactions, main effects were reported. Whenever an obstacle height main effect was found, a *post hoc* analysis was performed using a polynomial test to determine the linear trend. If significant interactions between the main factors were found, pair-wise between-group comparisons were performed using an independent *t*-test for each obstacle height, and a *post hoc* trend analysis was performed to determine the linear trend for each group. Results of the *P*-values, *T*-values, *F*-values and effect sizes in terms of partial η^2^ were reported. A significance level of α = 0.05 was set for all tests. The multi-link system approach (Hsu et al., 2016; Chien and Lu, 2017; Wu et al., 2019a) was used to synthesize the significant kinematic changes of individual joints and end-points to reveal the kinematic strategies of obstacle-crossing in the TCC group.

### Sample size calculation

An *a priori* power analysis based on pilot data of the pelvic orientations and lower limb joint angles from four subjects for each group using G*POWER (Erdfelder et al., 1996) for two-way mixed-design analysis of variance (ANOVA) for the comparisons of the angles with one between-subject factor (group) and one within-subject factor (obstacle height) determined that a projected sample size of 14 subjects for each group would be needed with a power of 0.8 and a large effect size (Cohen’s *d* = 0.63) at a significance level of 0.05. Thus, 15 subjects for each group were considered adequate for the main objectives of the current study.

## Results

### Subject demographics

Subject demographic data are given in Table 1. There were no significant between-group differences in age, sex, height, and mass.

**TABLE 1:** Means (standard deviations) of the demographic characteristic for the TCC (*n* = 15) and Control (*n* = 15) groups. *P*-values and *t*-values for between-group comparisons using independent *t*-tests are also given.

	TCC	Control	*t*-value	*p*-value
Age (years)	71.1 (5.4)	72.4 (6.1)	0.545	0.591
Body height (cm)	162.7 (6.7)	159.7 (5.6)	−1.199	0.243
Body mass (kg)	58.7 (6.5)	58.0 (10.4)	−0.188	0.852
Gender, number of				
females/males	4/11	4/11	–	–
TCC experience (years)	22.0 (10.5)	0	–	–

TCC, Tai-Chi Chuan.

### Temporal-spatial and end-point parameters

All the temporal-spatial and end-point parameters had no interactions between the group and height factors. The TCC group showed significantly greater leading (*p* = 0.025, partial η^2^ = 0.23) and trailing toe-obstacle clearances (*p* = 0.025, partial η^2^ = 0.22) but smaller trailing stride lengths (*p* = 0.01, partial η^2^ = 0.25) and heel-obstacle distance (*p* = 0.003, partial η^2^ = 0.28) when compared to the Control group (Table 2). No significant between-group differences were found in crossing speed, leading stride lengths, toe-obstacle distances and leading heel-obstacle distance (Table 2).

**TABLE 2:** Means (standard deviations, SD) of the crossing speed, stride length, and end-point parameters in the TCC and Control groups when crossing obstacles of different heights.

Variables	Obstacle height (%LL)	TCC	Control	*F*-values	Main effects	Partial eta squared
Crossing speed (m/s)	10	0.70 (0.05)	0.80 (0.14)	*F*_*G*_ = 4.050 *F*_*H*_ = 58.878	*P*_*G*_ = 0.115 *P*_*H*_ < 0.001**↓**	0.12 0.73
	20	0.65 (0.08)	0.71 (0.11)			
	30	0.60 (0.08)	0.64 (0.11)			
Leading stride length (% LL)	10	134.2 (8.0)	139.5 (11.7)	*F*_*G*_ = 0.997 *F*_*H*_ = 8.336	*P*_*G*_ = 0.328 *P*_*H*_ < 0.001↓	0.04 0.26
	20	133.8 (8.4)	136.4 (11.1)			
	30	132.6 (8.3)	134.9 (12.1)			
Leading toe-obstacle distance (% LL)	10	87.63 (8.65)	88.43 (10.81)	*F*_*G*_ = 0.001 *F*_*H*_ = 1.130	*P*_*G*_ = 0.977 *P*_*H*_ = 0.331	0.01 0.04
	20	89.30 (8.07)	87.84 (10.14)			
	30	87.58 (8.05)	87.92 (10.74)			
Leading heel-obstacle distance (% LL)	10	16.69 (2.88)	18.94 (4.96)	*F*_*G*_ = 0.809 *F*_*H*_ = 6.160	*P*_*G*_ = 0.377 *P*_*H*_ = 0.004**↓**	0.03 0.19
	20	15.77 (3.45)	16.61 (4.13)			
	30	14.94 (4.28)	15.40 (4.72)			
Leading toe-obstacle clearance (mm)	10	172.1 (34.1)	138.8 (39.7)	*F*_*G*_ = 5.896 *F*_*H*_ = 5.837	*P*_*G*_ = 0.025* *P*_*H*_ = 0.006↑	0.23 0.23
	20	185.9 (28.3)	164.8 (23.3)			
	30	190.0 (28.3)	167.9 (34.4)			
Trailing stride length (% LL)	10	128.0 (7.7)	141.7 (11.8)	*F*_*G*_ = 7.834 *F*_*H*_ = 1.165	*P*_*G*_ = 0.010* *P*_*H*_ = 0.311	0.25 0.05
	20	128.6 (8.9)	139.1 (11.1)			
	30	129.2 (10.7)	137.3 (10.9)			
Trailing toe-obstacle distance (% LL)	10	25.35 (4.69)	24.65 (4.41)	*F*_*G*_ = 0.295 *F*_*H*_ = 2.558	*P*_*G*_ = 0.592 *P*_*H*_ = 0.087	0.01 0.09
	20	26.04 (4.59)	24.36 (4.40)			
	30	24.46 (4.58)	24.11 (4.94)			
Trailing heel-obstacle distance (%LL)	10	72.46 (6.33)	85.90 (8.90)	*F*_*G*_ = 10.324 *F*_*H*_ = 0.621	*P*_*G*_ = 0.003* *P*_*H*_ = 0.541	0.28 0.02
	20	75.66 (6.45)	83.69 (8.43)			
	30	75.31 (8.95)	81.15 (10.61)			
Trailing toe-obstacle clearance (mm)	10	164.5 (30.4)	131.0 (44.2)	*F*_*G*_ = 5.857 *F*_*H*_ = 0.748	*P*_*G*_ = 0.025* *P*_*H*_ = 0.479	0.22 0.03
	20	179.3 (43.1)	134.5 (49.8)			
	30	173.9 (36.5)	145.4 (54.9)			

LL, leg length; *F_G_, f*-value for subject group; *F_H_, f*-value for obstacle height; *P_G_, p*-value for subject group; *P_H_, p*-value for obstacle height; * indicates a significant group effect (*P_G_* < 0.05); ↑ indicates a linearly increasing trend and ↓ indicates a linearly decreasing trend (*P_H_* < 0.05).

### Group effects on crossing angles

**FIGURE 2:**
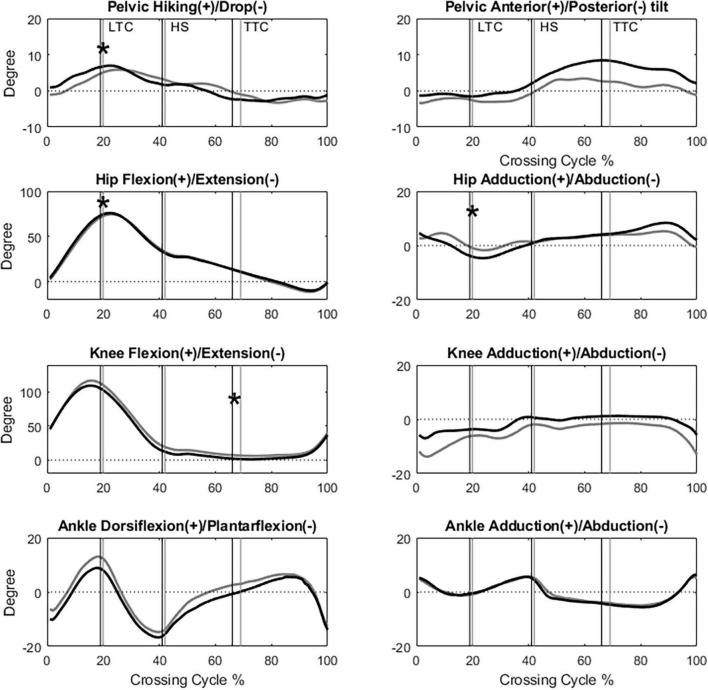
The angles of the pelvis, hip, knee and ankle joints of the leading limb in the sagittal and frontal plane of typical subjects of the TCC (black) and Control (gray) groups when crossing obstacles of 30% of leg length (LTC, leading toe above the obstacle; HS, heel-strike of the leading limb; TTC, trailing toe above the obstacle; *: Significant main group effect, *p* < 0.05).

**FIGURE 3:**
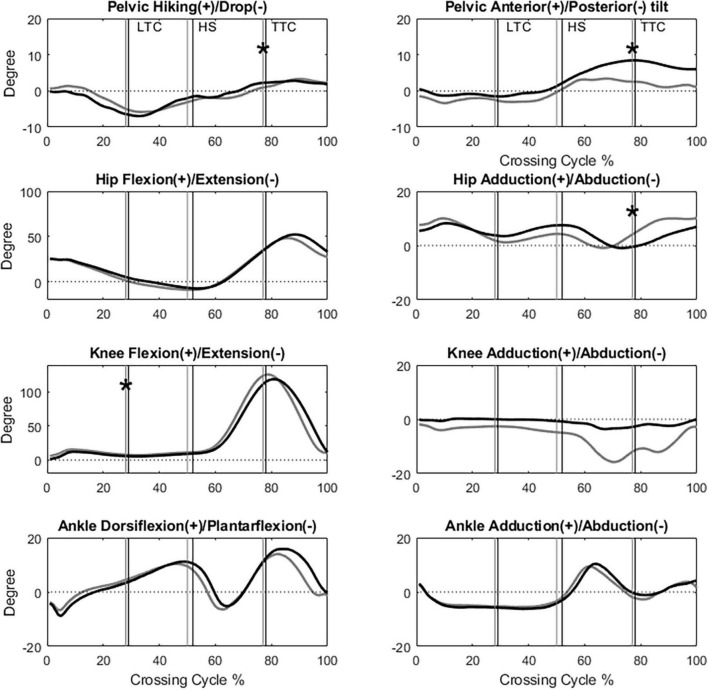
The angles of the pelvis, hip, knee and ankle joints of the trailing limb in the sagittal and frontal plane of typical subjects of the TCC (black) and Control (gray) groups when crossing obstacles of 30% of leg length (LTC, leading toe above the obstacle; HS, heel-strike of the leading limb; TTC, trailing toe above the obstacle; *: Significant main group effect, *p* < 0.05).

**FIGURE 4:**
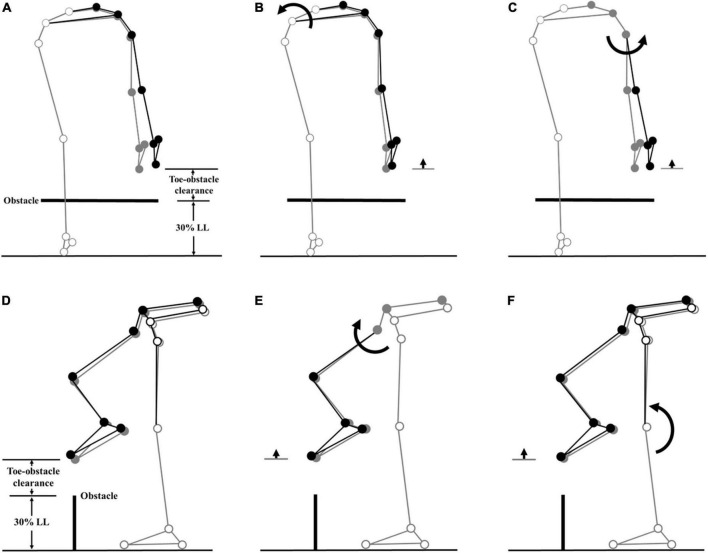
Effects of the observed significant angular changes at individual joints on the leading toe-obstacle clearance in the TCC group (black stick figure) compared with the Control group (gray stick figure) when the leading toe was above an obstacle of 30% LL in height. The stick model was drawn using each group’s marker positions of a typical subject, the segments with solid gray circles being joints of the reference limb. With the stance foot fixed to the ground, only one joint was rotated at a time according to the mean angular change reported in Tables 3, 4, while keeping the angles of the other joints fixed, and the segments of the stance limb and the segments of the swing limb distal to the current joint stationary. In the frontal plane, the observed increase of toe-obstacle clearance in the TCC group **(A)** was associated with significantly increased pelvic hiking **(B)** and decreased swing hip adduction **(C)**, while in the sagittal plane, the observed increase of toe-obstacle clearance **(D)** was associated with the significantly increased swing hip flexion **(E)** and decreased stance knee flexion **(F)**.

**FIGURE 5:**
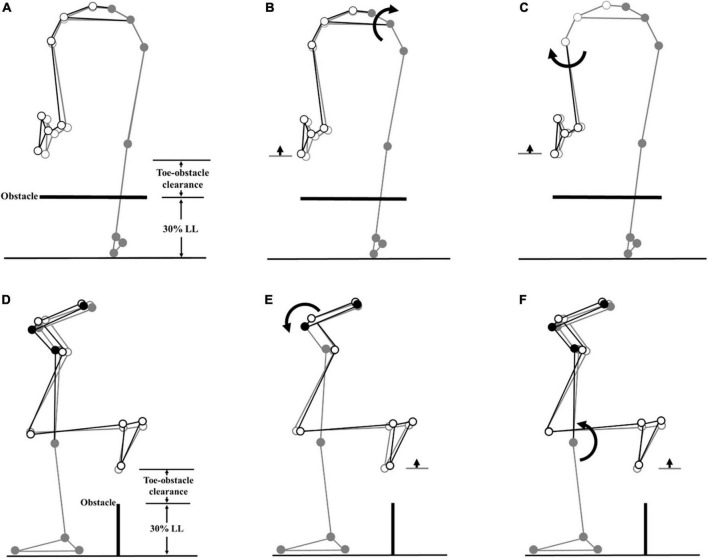
Effects of the observed significant angular changes at individual joints on the trailing toe-obstacle clearance in the TCC group (black stick figure) compared with the non-TCC controls (gray stick figure) when the trailing toe was above an obstacle of 30% LL in height. The stick model was drawn using each group’s marker positions of a typical subject, the segments with solid gray circles being joints of the reference limb. With the stance foot fixed to the ground, only one joint was rotated at a time according to the mean angular change reported in Tables 3, 5, while keeping the angles of the other joints fixed, and the segments of the stance limb and the segments of the swing limb distal to the current joint stationary. In the frontal plane, the observed increase of toe-obstacle clearance in the TCC group **(A)** was associated with significantly increased pelvic hiking **(B)** and decreased swing hip adduction **(C)**, while in the sagittal plane, the observed increase of toe-obstacle clearance **(D)** was associated with the significantly increased pelvic anterior tilt **(E)** and decreased stance knee flexion **(F)**.

All the kinematic variables had no interactions between the group and height factors. When the leading toe was above the obstacle, compared to the Control group, the TCC group showed significantly increased swing-side pelvic hiking (*p* = 0.048, partial η^2^ = 0.19) and swing hip flexion (*p* = 0.033, partial η^2^ = 0.21) but decreased swing hip adduction (*p* = 0.032, partial η^2^ = 0.17) and stance knee flexion (*p* < 0.032, partial η^2^ > 0.22) (Tables 3, 4 and Figure 2). When the trailing toe was above the obstacle, the TCC group showed significantly increased swing-side pelvic hiking (*p* = 0.008, partial η^2^ = 0.28) and anterior tilt (*p* = 0.02, partial η^2^ = 0.22) but decreased hip adduction (*p* = 0.021, partial η^2^ = 0.20) in the swing limb and knee flexion in the stance limb (*p* < 0.017, partial η^2^ > 0.22) (Tables 3, 5 and Figure 3). The observed significant angular changes of the pelvis and individual joints in the TCC group all contributed to the increased leading and trailing toe-obstacle clearances (Figures 4, 5).

**TABLE 3:** Means (standard deviations) of the crossing angles of the pelvis relative to the global in TCC practitioners (TCC) and non-TCC controls (Control) when the leading or trailing toe was above the obstacles of heights of 10, 20, and 30% of the subjects’ leg length (LL).

Variables	Group	Obstacle height (%LL)			*F*-values	Main effects	Partial eta squared
		10%	20%	30%			
**Leading toe above the obstacle**							
Hiking	TCC	3.8 (0.9)	5.9 (2.0)	9.1 (1.3)	*F*_*G*_ = 4.899; *F*_*H*_ = 40.99	*P*_*G*_ = 0.048*; *P*_*H*_ < 0.001↑	0.19; 0.70
	Control	3.2 (1.3)	5.5 (1.1)	6.9 (3.0)			
Anterior tilt	TCC	−0.9 (3.1)	−1.9 (3.1)	−3.2 (2.7)	*F*_*G*_ = 2.186; *F*_*H*_ = 40.89	*P*_*G*_ = 0.153; *P*_*H*_ < 0.001↓	0.09; 0.65
	Control	−1.8 (2.4)	−3.6 (2.5)	−5.2 (2.7)			
**Trailing toe above the obstacle**							
Hiking	TCC	−0.2 (1.3)	1.6 (1.6)	3.5 (1.8)	*F*_*G*_ = 8.548; *F*_*H*_ = 87.73	*P*_*G*_ = 0.008*; *P*_*H*_ < 0.001↑	0.28; 0.80
	Control	−1.8 (1.4)	−0.1 (1.4)	1.9 (1.8)			
Anterior tilt	TCC	4.1 (2.3)	5.8 (1.9)	8.4 (3.0)	*F*_*G*_ = 6.078; *F*_*H*_ = 90.13	*P*_*G*_ = 0.02*; *P*_*H*_ < 0.001↑	0.22; 0.80
	Control	1.5 (3.1)	3.1 (3.5)	5.4 (3.1)			

*F_G_, f*-value for subject group; *F_H_, f*-value for obstacle height; *P_G_, p*-value for subject group; *P_H_, p*-value for obstacle height; * indicates a significant group effect (*P_G_* < 0.05); ↑ indicates a linearly increasing trend and ↓ indicates a linearly decreasing trend (*P_H_* < 0.05). Hiking indicates that the ipsilateral hip joint center is higher than the contralateral hip.

**TABLE 4:** Means (standard deviations) of the crossing angles of the hip, knee and ankle joints of the leading swing limb and trailing stance limb in TCC practitioners (TCC) and non-TCC controls (Control) when the leading toe was above the obstacle of heights of 10, 20, and 30% of subjects’ leg length (LL).

Variables	Group	Obstacle height (%LL)			*F*-values	Main effects	Partial eta squared
		10%	20%	30%			
**Leading swing limb**							
Hip flexion	TCC	58.3 (6.0)	68.1 (5.0)	74.4 (5.7)	*F*_*G*_ = 5.219 *F*_*H*_ = 146.2	*P*_*G*_ = 0.033* *P*_*H*_ < 0.001↑	0.21 0.88
	Control	52.4 (5.2)	63.7 (5.6)	70.0 (6.8)			
Hip adduction	TCC	1.4 (3.2)	−0.7 (2.5)	−3.4 (3.9)	*F*_*G*_ = 5.136 *F*_*H*_ = 55.76	*P*_*G*_ = 0.032* *P*_*H*_ < 0.001↓	0.17 0.69
	Control	7.4 (7.4)	4.0 (7.6)	0.3 (7.6)			
Knee flexion	TCC	82.2 (7.5)	96.7 (9.2)	107.1 (5.5)	*F*_*G*_ = 2.425 *F*_*H*_ = 180.9	*P*_*G*_ = 0.134 *P*_*H*_ < 0.001↑	0.10 0.89
	Control	85.0 (6.3)	101.2 (6.6)	110.7 (5.7)			
Knee adduction	TCC	−4.7 (3.7)	−4.1 (3.7)	−3.8 (4.8)	*F*_*G*_ = 2.437 *F*_*H*_ = 10.89	*P*_*G*_ = 0.132 *P*_*H*_ < 0.001↑	0.09 0.32
	Control	−10.2 (6.2)	−7.8 (6.5)	−4.1 (7.2)			
Ankle dorsiflexion	TCC	10.6 (3.8)	12.1 (5.7)	8.7 (14.0)	*F*_*G*_ = 0.186 *F*_*H*_ = 0.797	*P*_*G*_ = 0.670 *P*_*H*_ = 0.456	0.01 0.03
	Control	9.8 (5.1)	11.6 (5.7)	13.0 (6.1)			
Ankle adduction	TCC	0.1 (3.2)	−1.0 (3.1)	−0.9 (3.0)	*F*_*G*_ = 0.065 *F*_*H*_ = 5.372	*P*_*G*_ = 0.801 *P*_*H*_ = 0.008↓	0.01 0.17
	Control	−0.3 (2.7)	−1.3 (2.8)	−1.1 (3.8)			
**Trailing stance limb**							
Hip flexion	TCC	4.1 (4.5)	3.8 (2.4)	2.9 (4.6)	*F*_*G*_ = 1.361 *F*_*H*_ = 1.422	*P*_*G*_ = 0.255 *P*_*H*_ = 0.251	0.05 0.06
	Control	2.1 (3.6)	1.9 (4.2)	1.7 (3.8)			
Hip adduction	TCC	6.7 (2.2)	5.4 (2.2)	2.2 (2.8)	*F*_*G*_ = 1.560 *F*_*H*_ = 43.27	*P*_*G*_ = 0.225 *P*_*H*_ < 0.001↓	0.07 0.66
	Control	5.5 (2.5)	3.7 (3.4)	1.3 (3.2)			
Knee flexion	TCC	4.9 (4.5)	5.0 (4.2)	4.8 (3.9)	*F*_*G*_ = 5.338 *F*_*H*_ = 0.112	*P*_*G*_ = 0.032* *P*_*H*_ = 0.894	0.22 0.89
	Control	8.6 (3.2)	8.5 (3.2)	8.3 (3.5)			
Knee adduction	TCC	0.1 (1.3)	−0.1 (1.3)	−0.5 (1.5)	*F*_*G*_ = 3.390 *F*_*H*_ = 2.064	*P*_*G*_ = 0.080 *P*_*H*_ = 0.140	0.14 0.09
	Control	−1.5 (2.4)	−1.4 (2.3)	−1.7 (1.9)			
Ankle dorsiflexion	TCC	5.7 (3.8)	6.1 (3.4)	2.3 (9.1)	*F*_*G*_ = 0.311 *F*_*H*_ = 4.893	*P*_*G*_ = 0.582 *P*_*H*_ = 0.011↓	0.01 0.16
	Control	6.7 (3.0)	5.1 (2.7)	4.5 (2.9)			
Ankle adduction	TCC	−6.5 (2.7)	−6.7 (2.8)	−5.7 (3.1)	*F*_*G*_ = 0.732 *F*_*H*_ = 0.884	*P*_*G*_ = 0.400 *P*_*H*_ = 0.419	0.03 0.03
	Control	−5.5 (2.2)	−5.5 (2.3)	−5.4 (4.1)			

*F_G_, f*-value for subject group; *F_H_, f*-value for obstacle height; *P_G_, p*-value for subject group; *P_H_, p*-value for obstacle height; * indicates a significant group effect (*P_G_* < 0.05); ↑ indicates a linearly increasing trend and ↓ indicates a linearly decreasing trend (*P_H_* < 0.05).

**TABLE 5:** Means (standard deviations) of the crossing angles of the hip, knee and ankle joints of the trailing swing limb and leading stance limb in TCC practitioners (TCC) and non-TCC controls (Control) when the trailing toe was above the obstacle of heights of 10, 20, and 30% of subjects’ leg length (LL).

Variables	Group	Obstacle height (%LL)			*F*-values	Main effects	Partial eta squared
		10%	20%	30%			
**Trailing swing limb**							
Hip flexion	TCC	27.3 (3.6)	32.2 (4.8)	36.5 (7.3)	*F*_*G*_ = 1.793 *F*_*H*_ = 54.65	*P*_*G*_ = 0.192 *P*_*H*_ < 0.001↑	0.07 0.68
	Control	24.5 (6.0)	29.9 (5.7)	33.9 (6.0)			
Hip adduction	TCC	1.5 (2.7)	1.0 (3.8)	0.1 (4.6)	*F*_*G*_ = 6.118 *F*_*H*_ = 0.669	*P*_*G*_ = 0.021* *P*_*H*_ = 0.517	0.20 0.03
	Control	4.0 (3.1)	5.2 (5.1)	4.9 (6.2)			
Knee flexion	TCC	92.7 (3.2)	109.0 (8.6)	120.3 (8.0)	*F*_*G*_ = 2.581 *F*_*H*_ = 125.1	*P*_*G*_ = 0.122 *P*_*H*_ < 0.001↑	0.11 0.85
	Control	96.8 (7.4)	112.0 (9.0)	124.4 (7.1)			
Knee adduction	TCC	−5.0 (4.0)	−5.7 (3.7)	−4.6 (3.8)	*F*_*G*_ = 34.885 *F*_*H*_ = 1.860	*P*_*G*_ = 0.136 *P*_*H*_ = 0.170	0.12 0.09
	Control	−10.3 (7.0)	−8.3 (7.2)	−7.6 (6.7)			
Ankle dorsiflexion	TCC	2.9 (7.1)	8.0 (7.9)	8.4 (11.5)	*F*_*G*_ = 0.010 *F*_*H*_ = 19.89	*P*_*G*_ = 0.920 *P*_*H*_ < 0.001↑	0.01 0.43
	Control	2.5 (7.5)	7.0 (9.0)	10.8 (7.8)			
Ankle adduction	TCC	0.1 (3.8)	−1.0 (4.8)	−1.5 (4.3)	*F*_*G*_ = 1.237 *F*_*H*_ = 3.223	*P*_*G*_ = 0.276 *P*_*H*_ = 0.048↓	0.05 0.11
	Control	−2.0 (2.8)	−2.3 (3.6)	−2.5 (3.3)			
**Leading stance limb**							
Hip flexion	TCC	13.3 (5.2)	12.9 (5.2)	13.3 (7.6)	*F*_*G*_ = 1.559 *F*_*H*_ = 0.710	*P*_*G*_ = 0.223 *P*_*H*_ = 0.496	0.06 0.03
	Control	11.4 (4.1)	10.4 (4.0)	10.9 (4.6)			
Hip adduction	TCC	8.7 (3.7)	7.9 (4.2)	4.2 (5.3)	*F*_*G*_ = 0.184 *F*_*H*_ = 51.125	*P*_*G*_ = 0.671 *P*_*H*_ < 0.001↓	0.01 0.66
	Control	10.7 (4.0)	8.2 (4.2)	4.0 (5.3)			
Knee flexion	TCC	5.6 (6.6)	2.9 (7.4)	1.9 (9.0)	*F*_*G*_ = 6.608 *F*_*H*_ = 20.47	*P*_*G*_ = 0.017* *P*_*H*_ < 0.001↓	0.22 0.47
	Control	11.6 (4.6)	8.9 (3.4)	7.6 (3.1)			
Knee adduction	TCC	0.6 (1.1)	0.6 (1.5)	0.8 (2.2)	*F*_*G*_ = 4.138 *F*_*H*_ = 3.895	*P*_*G*_ = 0.055 *P*_*H*_ = 0.028↑	0.17 0.16
	Control	−1.6 (2.5)	−1.0 (2.3)	−0.5 (2.5)			
Ankle dorsiflexion	TCC	3.1 (3.0)	3.6 (2.7)	−1.5 (11.3)	*F*_*G*_ = 1.545 *F*_*H*_ = 3.759	*P*_*G*_ = 0.225 *P*_*H*_ = 0.030↓	0.06 0.13
	Control	4.0 (3.0)	3.8 (3.3)	2.9 (4.5)			
Ankle adduction	TCC	−4.4 (2.6)	−5.3 (3.2)	−4.2 (2.6)	*F*_*G*_ = 0.077 *F*_*H*_ = 1.065	*P*_*G*_ = 0.784 *P*_*H*_ = 0.352	0.01 0.04
	Control	−4.5 (2.2)	−4.3 (3.0)	−4.3 (2.8)			

*F_G_, f*-value for subject group; *F_H_, f*-value for obstacle height; *P_G_, p*-value for subject group; *P_H_, p*-value for obstacle height; * indicates a significant group effect (*P_G_* < 0.05); **↑** indicates a linearly increasing trend and ↓ indicates a linearly decreasing trend (*P_H_* < 0.05).

### Height effects on crossing angles

With increasing obstacle height, both TCC and Control groups linearly reduced the crossing speeds, leading stride length, leading toe-obstacle distance, leading heel-obstacle distance, and trailing heel-obstacle distance but linearly increased the leading toe-obstacle clearance (*p* < 0.01, partial η^2^ > 0.19, Table 2). When the leading toe was above the obstacle, both groups linearly increased swing-side pelvic hiking and posterior tilt, hip flexion, knee flexion, and ankle abduction of the swing limb, but linearly decreased the hip adduction and knee abduction in the swing limb, as well as decreased the ankle dorsiflexion of the stance limb (*p* < 0.02, partial η^2^ > 0.16, Tables 3, 4). On the other hand, when the trailing toe was above the obstacle, both groups linearly increased swing-side pelvic hiking and anterior tilt, hip flexion, knee flexion, ankle dorsiflexion and abduction of the swing limb, and knee adduction of the stance limb, as well as linearly decreased the hip adduction, knee flexion and ankle dorsiflexion of the stance limb (*p* < 0.05, partial η^2^ > 0.11, Tables 3, 5).

## Discussion

The current study aimed to identify the kinematic strategies of the pelvis-leg apparatus in older long-term TCC practitioners during obstacle crossing, an activity of daily living that exposes older people to a higher risk of falls (Tinetti and Speechley, 1989; McFadyen and Prince, 2002). Compared to the Control group, the TCC group showed increased leading and trailing toe-obstacle clearances *via* a specific multi-joint kinematic strategy involving the pelvis, the hip of the swing limb and the knee of the stance limb, which appeared to be related to the training characteristics of TCC movements. The current results suggest that with the specific kinematic strategy, the older individuals with TCC experience for thirteen or more years had reduced tripping risks compared to their non-TCC peers. Further study may be needed to determine the period of TCC practice required to have similar benefits in older people.

During leading-limb crossing, the TCC group increased the leading toe-obstacle clearance, an indication of reduced risk of tripping (Sparrow et al., 1996), with a kinematic strategy consisting of increased swing-side pelvic hiking and swing hip flexion and decreased swing hip adduction and stance knee flexion. The TCC group also increased the trailing toe-obstacle clearance by increasing the swing-side pelvic hiking and anterior tilt and decreasing the swing hip adduction and stance knee flexion. All the significant pelvic and joint kinematic changes contributed to the increase of the toe-obstacle clearances. This is in contrast to kinematic changes found in patient populations such as patients with type II diabetes mellitus (Hsu et al., 2016), isolated posterior cruciate ligament deficiency (Kuo et al., 2017), and severe idiopathic thoracic scoliosis (Wu et al., 2019a), in which some kinematic changes were tending to increase the toe-obstacle clearance while others showed the opposite effects. Such antagonistic kinematic changes may be interpreted as some kinematic changes with positive effects (potential upward deviations of the end-point) were needed to compensate for the negative (decreasing) effects on toe-obstacle clearance of some joint kinematic deviations. The TCC group appeared to have acquired a synergistic multi-joint kinematic strategy in which each kinematic change contributed positively to the observed increase of toe-obstacle clearance.

In the kinematic strategy adopted by the TCC group, the motion of the pelvis was the main contributor to the increased toe-obstacle clearance, especially the pelvic hiking that lifted the swing limb and the end-point directly. Considering the end-point as the tip of a pendulum rotating about a joint, being proximal to the hip and knee, the pelvic anterior tilt also had a greater effect on the position of the end-point and thus the toe-obstacle clearance (Chen et al., 2016; Wu et al., 2019a). On the other hand, the kinematic changes at the swing hip and stance knee appeared to provide refinement for precision control of the end-point. Overall, the observed synergistic multi-joint kinematic strategy in older long-term TCC practitioners was also accompanied by more in-phase but less variable inter-joint coordination patterns in the lower limbs during obstacle crossing previously found, compared to older people without TCC training (Kuo et al., 2021b).

The altered motions of the pelvis involved in the synergistic multi-joint kinematic strategy in older long-term TCC practitioners did not seem to cause adverse effects on the balance control during obstacle-crossing as previously reported in older people without TCC experience (Hsu et al., 2016; Kuo et al., 2017; Wu et al., 2019a). While the increased swing-side hiking and an anterior tilt of the pelvis increased the toe-obstacle clearance, they would also potentially affect the motions of the trunk and thus the body’s centre of mass (COM), leading to perturbations to whole-body posture and balance with increased risk of loss of balance, another major contributing factor to falls in older people (Overstall et al., 1977; Blake et al., 1988; Campbell et al., 1990). This is especially critical for leading-limb crossing as the neuromechanical challenges for balance control are greater than trailing-limb crossing when the body moves away from the trailing stance limb (Wu et al., 2020). Failure to recover balance would lead to falls once a trip or loss of balance occurs. For the TCC group, in contrast to older people without TCC experience, the body posture changes led to increased leading toe-obstacle clearance and reduced anterior COM-COP IA with better stability (Kuo et al., 2022). Moreover, the long-term TCC practitioners were found to show a well-controlled, more conservative COM-COP motion for a smoother and more stable bodyweight transfer (Hahn and Chou, 2004; Wang et al., 2010b) with relatively less mechanical energy expenditure than older peers (Kuo et al., 2021a) *via* a less variable lower-limb inter-joint coordination (Kuo et al., 2021b). A similar phenomenon was also found during trailing-limb crossing. The results of the current and previous studies suggest that long-term older TCC practitioners cross obstacles with a lesser risk of tripping and loss of balance than non-TCC practitioners *via* the specific kinematic strategy.

The kinematic strategy in the end-point and pelvis-leg motion control observed in the current long-term TCC practitioners during obstacle-crossing appeared to be related to the training characteristics of TCC movements, which were all helpful for increasing the lower limb muscle strength and body flexibility, improving whole-body balance, and reducing the variability of the inter-joint coordination of the lower limbs during obstacle-crossing (Jacobson et al., 1997; Lan et al., 1998; Wu, 2002; Mak and Ng, 2003; Kuo et al., 2021b). TCC practice is also helpful for improving attention, memory, executive functions and motor planning (Wong et al., 2009; Man et al., 2010; Lv et al., 2022). Long-term practice of TCC was found to help improve memory (Yue et al., 2020) and sensory organization in postural and balance control (Wong et al., 2001, 2009; Wu, 2002; Wu et al., 2002; Mak and Ng, 2003; Tsang et al., 2004). The positive effects of TCC practice are beneficial to the performance of obstacle-crossing, which requires not only physical strength but also the ability of motor planning (Clark et al., 2014) and an adequate allocation of cognitive resources, including memory (Patla and Vickers, 1997; Mohagheghi et al., 2004), attention and executive function (Clark et al., 2014; Clark, 2015). A recent study showed that obstacle-crossing kinematic features in TCC were found to be Central Nervous System (CNS) controlled *via* an obstacle-height independent best-compromise strategy between the conflicting objectives of minimizing mechanical energy expenditure and maximizing the foot-obstacle clearance (Kuo et al., 2021a). It appears that long-term older TCC practitioners benefited from the positive effects of long-term TCC practice both physically and neurologically, contributing to the formation of the observed specific synergistic multi-joint kinematic strategy for crossing obstacles with reduced risks of tripping and loss of balance.

The current study was the first to identify the kinematic strategies of the pelvis-leg apparatus during obstacle-crossing in long-term older TCC practitioners. Further study on the kinetics of the lower limb joints and the associated muscular activities *via* EMG analysis may be needed to provide more insight into the neuromechanical control involved in the observed kinematic strategy. The current study was also limited to a cross-sectional design as studies before and after TCC training cannot be used in TCC with thirteen years of experience. However, the current results provide important evidence to encourage further longitudinal studies on the multi-joint kinematic strategies before and after short-term TCC training, which will help identify the specific causal effects of TCC training on the postural adjustments during obstacle-crossing in the older population.

The current results have several possible clinical implications and applications. Older people may benefit from the positive effects of long-term TCC practice both physically and neurologically, which helps to form the observed synergistic multi-joint kinematic strategy for obstacle-crossing. Knowledge of the similarities or differences in the control strategies and the resulting joint mechanics between TCC and non-TCC groups would be helpful in developing improved fall prevention strategies and for making better use of TCC training in fitness programs for the elderly. It is suggested that TCC practice may be adjusted and integrated into the daily activities of inpatient and long-term care facilities, which may reduce the risk of tripping when negotiating obstacles and improve the general physical condition of the older population.

## Conclusion

The current study identified the kinematic changes of the pelvis and the lower limb joints and, through a multi-link analysis, revealed a specific synergistic multi-joint kinematic strategy adopted to reduce tripping risks during obstacle-crossing in older long-term TCC practitioners as compared to non-TCC healthy controls. In such a strategy, each significant kinematic change contributed positively to the observed increase of leading and trailing toe-obstacle clearances. The current results suggest that the older long-term TCC practitioners had the necessary physical and neurological abilities for the observed kinematic strategies and the associated toe-obstacle clearances. Long-term TCC practice appeared to be helpful for older people in reducing the risk of tripping and the subsequent loss of balance.

## Data availability statement

The original contributions presented in this study are included in the article/supplementary material, further inquiries can be directed to the corresponding author.

## Ethics statement

The studies involving human participants were reviewed and approved by the China Medical University and Hospital Research Ethics Committee. The patients/participants provided their written informed consent to participate in this study.

## Author contributions

H-PH: conceptualization, data curation, resources, formal analysis, and writing–original draft. C-CK and S-HL: data curation, resources, and writing–original draft. S-CC: data curation, formal analysis, and writing–original draft. T-JH: data curation, resources, and writing–original draft. T-WL: conceptualization, supervision, writing–original draft, and writing–review and editing. All authors contributed to the article and approved the submitted version.
